# A New Adhesive Plaster

**Published:** 1875-10

**Authors:** 


A New Adhesive Plaster.—Kauvin recommends a mixture of twenty parts of mucilage of gum arabic and one part glycerine, spread three or four times upon linen at sufficient intervals to allow it to thoroughly dry. This plaster is glassy, pliable, does not deteriorate with keeping, and is considerably cheaper than English plaster or diachylon plaster.—*The Druggists' Circular.*

